# Development of the prototype concise safe systems checklist tool for general practice

**DOI:** 10.1186/s12913-020-05396-y

**Published:** 2020-06-16

**Authors:** Ian Litchfield, Rachel Spencer, Brian G. Bell, Anthony Avery, Katherine Perryman, Kate Marsden, Sheila Greenfield, Stephen Campbell

**Affiliations:** 1 Institute of Applied Health Research, College of Medical and Dental Sciences, University of Birmingham, Birmingham, UK; 2 Unit of academic primary care, Warwick Medical School, University of Warwick, Coventry, UK; 3 Division of Primary Care, School of Medicine, University of Nottingham, Nottingham, UK; 4 Division of Population Health, Health Services Research and Primary Care, hester Patient Safety Translational Research Centre, School for Health Sciences, University of Manchester, Manchester, UK

**Keywords:** Patient safety, Checklist, General practice, Quality indicators healthcare

## Abstract

**Background:**

In the course of producing a patient safety toolkit for primary care, we identified the need for a concise safe-systems checklist designed to address areas of patient safety which are under-represented in mandatory requirements and existing tools. This paper describes the development of a prototype checklist designed to be used in busy general practice environments to provide an overview of key patient safety related processes and prompt practice wide-discussion.

**Methods:**

An extensive narrative review and a survey of world-wide general practice organisations were used to identify existing primary care patient safety issues and tools. A RAND panel of international experts rated the results, summarising the findings for importance and relevance. The checklist was created to include areas that are not part of established patient safety tools or mandatory and legal requirements. Four main themes were identified: information flow, practice safety information, prescribing, and use of IT systems from which a 13 item checklist was trialled in 16 practices resulting in a nine item prototype checklist, which was tested in eight practices. Qualitative data on the utility and usability of the prototype was collected through a series of semi-structured interviews.

**Results:**

In testing the prototype four of nine items on the checklist were achieved by all eight practices. Three items were achieved by seven of eight practices and two items by six of eight practices. Participants welcomed the brevity and ease of use of the prototype, that it might be used within time scales at their discretion and its ability to engage a range of practice staff in relevant discussions on the safety of existing processes. The items relating to prescribing safety were considered particularly useful.

**Conclusions:**

As a result of this work the concise patient safety checklist tool, specifically designed for general practice, has now been made available as part of an online Patient Safety Toolkit hosted by the Royal College of General Practitioners. Senior practice staff such as practice managers and GP partners should find it a useful tool to understand the safety of less explored yet important safety processes within the practice.

## Background

The importance of patient safety continues to be recognised yet progress on improvement has been modest and patients everywhere continue to experience avoidable harm and substandard care [1]. One setting in the UK where patients experience increasing risk is primary care; where the diversity, scope and variation in infrastructure is combined with unprecedented demand from an ageing and chronically ill population [2]. To help meet this need the National Institute for Health Research School for Primary Care Research (UK) (NIHR-SPCR) funded the development of a multi-strand Patient Safety Toolkit (PST) comprising a number of tools that would equip practices to independently address a range of patient safety issues [3]. Our steering panel of experts felt that checklists are an important patient safety tool that remains under-utilised in primary care settings. Originally used in highly ordered environments such as the aviation industry [4] they have been effectively adopted in secondary care where patient safety has been improved in a range of specialities such as surgery, haemodialysis, anaesthesiology and other highly protocol driven areas of medicine [4–7].

Limited attempts have been made to transfer the success of checklists to the diverse environment of primary care. For example, the NHS Education for Scotland (NES) checklist [8] is a 78 item tool designed to meet regulatory compliance, or the checklists designed by UK indemnity organisations and made available to general practices to assist in medico-legal risk assessments [9, 10]. However these examples are either lengthy [8], not specific to patient safety [8, 9] or require external facilitation [9]. An approach that had not yet been taken was the development of a concise or ‘short-form’ checklist, specific to patient safety within UK general practice and designed to be completed by practice staff without external support.

The aim of this work was to produce a precise checklist that would minimise the impact on staff time and frame constructive discussions that reconsider and if needed improve existing safety related processes [3]. It would also have the potential to address areas of the patient safety taxonomy developed for the PST not covered by other tools [11]. The items included on the checklist were to be informed by international expert consensus [12], avoiding issues already being met by mandatory health and safety requirements [13, 14] and complementing the content of other tools in the Patient Safety Toolkit for UK General Practices [12, 15, 16]. Here we describe the development of the prototype safe-systems checklist, (PSC) including the rationale of the final design and the quantitative and qualitative testing of the prototype within UK general practice.

## Methods

**Fig. 1:**
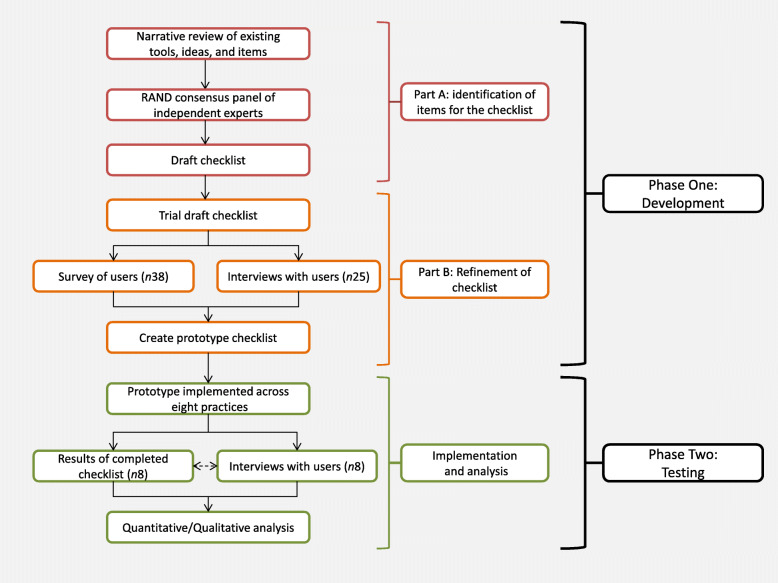
Flow chart showing study design

The National Institute of Health Research (NIHR) School for Primary Care Research (SPCR) Patient Safety Toolkit for general practices involved multiple academic centres (Birmingham, Exeter, Keele, Manchester, Nottingham, Oxford and Southampton) [10]. All participants were consented in line with the procedures of the Patient Safety Toolkit project [3]. Here we present the two discrete phases of the development of the prototype safe-systems checklist (PSC); Phase One consisted of the development of the (PSC which was conducted in two parts by senior members of the research team BB, RS, AA, and SC; In Phase Two the checklist was tested by practice staff at eight practices within North Staffordshire as part of the broader implementation of the patient safety toolkit [3]. All The overall study design is summarised in Fig. 1.

### Phase One: Development

Part A of the development phase consisted of the identification of items for the draft list and Part B the refinement of the draft list integrating user feedback to produce the final prototype checklist.

#### Part A

An extensive narrative review of tools for general practice patient safety was conducted by two of the authors (RS, SC) [11] and existing tools, ideas and items were considered by a Research and Development Corporation (RAND) consensus panel of nine international experts [12] who produced a score relating to the priority of each of the items with the nascent checklist formed from items deemed ‘high priority’. These were then compared to Care Quality Commission (CQC) requirements [13] and the Canadian Quality Book of Tools (CQBT) ‘safe theme’ [17] and items already included in these or other UK mandatory requirements [13, 14] were removed from the checklist in order to prevent duplication. Finally the wording of the draft list of items was reviewed by the study team for clarity and precision and those judged to be similar in theme were combined leaving 13 items.

#### Part B

The 13-item checklist was used by an opportunistic sample of staff from 16 practices recruited as part of the wider patient safety toolkit (PST) project [3, 15]. The checklist was completed by staff alongside a questionnaire which explored their perspectives on the items of the checklist primarily relating to their utility. Staff that took part in this initial testing were practice managers, and nurses reflecting the intention that it could be used by a range of clinical and non-clinical staff. Individuals were interviewed by KM using a topic guide which asked whether they felt individual items addressed important aspects of patient safety, those already being routinely addressed within the practice, and if they had made any changes to practice processes as a result of completing the checklist. Staff were also asked about the clarity of the items presented and if they were able to answer the questions posed as either ‘yes’ or ‘no’ as intended.

### Phase Two: Testing of the prototype safe-systems checklist

Phase Two involved the distribution and use of the PSC across a number of practices. Convenience sampling [18] was used to recruit eight practices previously involved in the PST project. Each participating practice was visited by the researcher (KM) who described how the checklist should be used and by whom i.e. that it could be completed by either clinical or non-clinical staff. The checklist was completed once by a member of staff chosen by the individual practice and the summary statistics of the compliance with each item by participating practices was produced.

Semi-structured interviews [19] were then conducted individually with the one member of practice staff at each of the eight practices that had used the checklist. The interviews were completed by an experienced qualitative researcher with expertise in primary care, patient safety and health service research (KM) who had not previously met any of the participants using a topic guide that asked questions on their expectations and experience of using the PSC including its usability and applicability, and how it might be improved. All were digitally recorded before being transcribed verbatim and nvivo used to manage the data. We used a post hoc deductive analysis based on the relevant domains and constructs of the consolidated framework for implementation research (CFIR) [20] and emergent sub-themes noted. To enable this a sample of three transcripts underwent an independent analysis by two of the authors (IL & SG). These analyses and any discrepancies were then discussed by both before the analysis of the remaining transcripts was conducted by IL. Our intention was not to reach data saturation but to gain a more structured understanding of user perceptions of the PSC. The ultimate interpretation of the data was approved by all authors.

## Results

### Phase One: Development

#### Part A – Identification of the items for inclusion on the draft checklist

A total of 205 items were identified from the literature review for consideration by the RAND consensus panel [12] who judged 37 to be of “high priority”. Following comparison with the CQC, CQBT and UK mandatory requirements and the review of the study team for repetition and clarity a total of 13 items were eventually included on the draft checklist.

#### Part B – Refinement of the draft checklist to produce the final prototype

A total of 10 East Midlands (EM) and six Greater Manchester (GM) practices tested the draft checklist. A representation of the characteristics of the practices participating in the toolkit project can be found in Table 1. Within these practices a total of 38 respondents (23 from EM and 15 from GM) completed the pilot version of the checklist and accompanying questionnaire (23 GPs, four nurses, nine practice managers, and two administrative staff). An additional 25 staff from participating practices were interviewed with regard to the applicability of the PST (three GP registrars, three practice nurses, six practice managers and 13 GPs) interviews lasted between 10 and 33 min.

**Table 1:** Summative characteristics of participating practices [10]

	List Size^**a**^	Under 18^**a**^	Over 65^**a**^	% Non-White^**b**^	Deprivation Score (based on IMD score [21])^**a**^	QOF Score (2013)^**a**^	% Female^**b**^
**Practice Average/SD**^**1**^	7363/2830	20.7% 3.1%	19.5% 3.3%	13.1% 15.2%	22.5 9.1	988.1 7.6	52.9% 3.8%
**English Average**	7041^a^	20.8%^a^	16.7%^a^	13%^b^	21.5^a^	961^a^	51%^b^
**Practice Median/IQR**^**1**^	6804 4327	19.7% 5.9%	20.9% 5.8%	1.6% 30.9%	17.8 22.2	989.4 10.2	52.4% 8.0%

^1^The practice average, standard deviation, median, and IQR use values that are weighted by the practice list size

^a^taken from the national general practice profiles (Public Health England) www.apho.org.uk/PRACPROF/

^b^taken from the GP patient survery July 2014 http://practicetool.gp-patient.co.uk/practice

At this stage the checklist was divided into four sections (information flow, safety information about the practice, prescribing, and use of IT systems), each with an introductory statement that was taken directly from our project taxonomy of patient safety [11].

A summary of the results from the development of the PSC can be found in Table 2 relating to one of the domains. Within each we define the domain, present the draft items, the rationale and evidence for each, the changes made as a result of the feedback collated from the questionnaires and semi-structured interviews and the related item as it appeared on the PSC which numbered nine in total.

**Table 2:** Development of Items for inclusion in prototype checklist

Development of draft checklist					Prototype Checklist
***Description***	***Item (s)***	***Rationale***	***Evidence***	***Refinement***	***Item***
Information flow The practice has a systems based approach to processing incoming results* and information in to and out of the practice, which prevents human and electronic error in data handling. *results = lab results, reports or investigations, and letters.	All incoming clinical information is seen by a GP in the practice to view and action before or after being filed, scanned or coded in the patient’s medical record.	The practice has a systems based approach to processing incoming results* and information into and out of the practice, which prevents human and electronic error in data handling [* results: lab results, reports or investigations]and letters) [12].	(PMCPA) [22] (Premises Records Equipment/Devices and Medicines management section) (http://www.rcgp-practiceaccreditation.org.uk	Wording changes - in order to allow practices to nominate appropriately trained staff to handle mail rather than just GPs	All incoming clinical information is seen by nominated members of the team trained (or with relevant clinical experience) to deal appropriately with this information before the information is filed in the patient’s record.
	Where an incoming result, report or investigation requires follow-up or a diarised activity, it is recorded in the patient’s medical record and acted upon [for example follow up of blood tests such as PSA, INR etc.]	Where an incoming result, report or investigation requires follow-up there are systems in place to ensure it occurs.	Adapted from PCMPA [22] and informed by Casalino et al. [23].	These items were combined as they were felt to be too similar	Where a clinician decides it is indicated, the patient (or where appropriate the patient’s representative) is informed of abnormal investigation results in an appropriately and timely manner and this contact is documented in the patient’s record.
	The patient (or where appropriate, families and carers) is informed of an abnormal investigation results in an appropriate and timely manner and this is documented in the patient’s record.	The provider has a written policy for informing patients, or where appropriate, families and carers, of the results of investigations and the policy is explained to them.	Adapted from PMCPA [22].		
	The practice keeps a record or log of their minor operations which will have the following information recorded; 1) date; 2) patient name; 3) procedure performed; 4) team members involved; 5) whether a specimen was sent for histology; 6) patient consent; 7) complications; 8) patient informed of result.	This log represents the basic safety information required about any surgery performed	Taken direct from PMCPA [22] (provider management), a template could easily be designed to collect this information		The practice keeps a log of minor operations
Safety information about the practice The practice has a systems based approach to supplying information about safety procedures required by permanent and temporary staff.	Up-to-date information on the practice policies and procedures, and local facilities and services is provided to guide locums and other temporary clinical staff who work in the premises, in the form of a clinical staff handbook (hard copy).	There is no current legislative requirement specifically directed at trainees or temporary staff.	Dutch consensus process exploring safe working conditions from locum staff [24].	Items combined as seen as too similar. Requirement for hard copy information was removed after discussion within our project team, considering the change to paper-light practices.	Up-to-date information on practice policies, procedures and local facilities/services is provided to guide all temporary clinical staff (including GP registrars).
	There is an up-to-date office procedure manual (hard copy and/or electronic copy) covering the administrative procedures and systems for the daily running of the practice to which team members have access. These policies are discussed and agreed by team members and are reviewed at least annually.	There is no central policy document of safety procedures readily available to all staff.	Review of factors supporting successful teamwork in primary care [25].		
Working with patients for safe prescribing The practice has a systems based approach to working with patients to improve the safety of prescribing practices.	The practice works with patients to ensure medication list accuracy (medication reconciliation) upon hospital referral.	No such process for medicine reconciliation exists despite the potential impact on patient safety.	Review of reconciliation issues [26, 27].	Removed as seen as being beyond practice’s control.	
	Non-collection of prescriptions held by the practice are monitored and followed-up by the practice and medications which are not claimed by patients are a trigger for review and audit in partnership with local pharmacies.^d^	Non-collection might reflect medication error, poor compliance or other patient safety issues.	A study of medication reviewing in primary care^27^ and is also included in PMCPA [22].	Wording changes - in order to simplify the item.	Non-collection of prescriptions is monitored or followed-up and is a trigger for review and audit in partnership with local pharmacies.
	Patients discharged from hospital should have a recorded follow up appointment with a member of the practice clinical team within 1 month.	Patients at high risk of patient safety incidents should be followed-up at risky care transitions.	Originally from a US process mapping study [28].	Clinicians believed that it was unrealistic to follow-up all of the discharges within one month so we added the word ‘vulnerable’ to this item.	Vulnerable patients discharged from hospital are followed-up by a member of the clinical team within 1 month.
IT indicators for prescribing The practice has fit for purpose IT systems for prescribing which work with prescribers to make prescribing a safer activity.	The practice uses an electronic prescribing system for all prescriptions (Computerized Physician Order Entry (CPOE)).	Drive to implement CPOE primarily comes from its presumed benefit in reducing medical errors.	Evidence of CPOE can reduce medication errors [29].	Removed - did not allow practices enough flexibility to serve patients.	
	Prescribers code the indication for the drug with each prescription using the electronic prescribing system (with the exception of topical medications without active ingredients).	This is good practice and there is currently no legislative requirement for it to be done.	Canadian study of electronic coding of prescription indication [30].	Wording changed for clarity.	The indication for all repeat medications is coded within the electronic record (excluding topical preparations).
	The practice has and uses, the most up-to-date alerting software available, routinely on all computers used for prescribing in relation to allergies and duplicates, drug-drug interactions, contraindications in terms of drug –disease, drug-age and potentially drug-lab value interactions.	The safety features of software systems are effective in alerting users about potential clinical hazards and errors during medication order entry.	Delphi study on electronic safety systems [31].	Felt to be imbedded in the computer systems.	
	All staff (including GPs) are trained to make safe use of the prescribing elements of their clinical IT systems.	Specific training in IT prescribing systems is not a mandatory requirement and yet is essential for all team members involved in prescribing.	Delphi study on electronic safety systems [31].	Wording changed for clarity.	All staff are trained to make safe use of the prescribing elements of the clinical IT system which are relevant to their role.

### Phase Two: Testing of the prototype safe-systems checklist

Eight participating practices within North Staffordshire (NS) agreed to test the checklist.

#### Quantitative data

Table 3 shows the percentage of practices which answered yes to final checklist items. Items with a response of ‘No’ indicate where as a practice they feel they have not addressed a checklist item and might need to make a change to its systems. The two items with the lowest percentage of ‘Yes’ responses (25% of practices did not think achieve these safety goals) were item 6 regarding the failure to monitor the non-collection of prescriptions and item 7 relating to follow-up of vulnerable patients following discharge from hospital. Several items were met by all participating practices including the appropriate handling of incoming clinical information and the timely follow-up of abnormal results.

**Table 3:** Percentage of “yes” answers across practices

Item Number	Summary description	% Yes answers
1	All incoming clinical information is seen by trained or clinically experienced members of staff before filing.	100
2	Where incoming clinical information requires follow-up this is documented in the patient’s record and acted upon.	87
3	Where a clinician decides it is indicated, the patient (or a suitable/ appropriate representative) is informed of abnormal investigation results and documented in the patient’s record.	100
4	The practice keeps a log of minor operations containing key information including, • Date/patient’s name • Procedure performed • Who performed the operation and who assisted • Any complications	87
5	Up-to-date information on practice policies, procedures and local facilities/services is provided to guide all temporary clinical staff (including GP registrars).	100
6	Non-collection of prescriptions is monitored and a trigger for review in partnership with local pharmacies.	75
7	Vulnerable patients discharged from hospital are followed-up by a member of the clinical team within 1 month.	75
8	The indication for repeat medications is coded within the electronic record.	87
9	Staff are trained to make safe use of the prescribing elements of the clinical IT system relevant to their role.	100

#### Qualitative data

We interviewed eight participants each from one of the practices that trialled the prototype checklist. Of these three were practice managers, three were general practitioners, one was healthcare assistant and one was a practice nurse manager. Interviews lasted between and 13 and 37 min [10].. The practices they represented were situated within a variety of socio-economic backgrounds represented using the Index of Multiple Deprivation [21] and patient list sizes from 4000 to just over 12,000 these characteristics are summarised in Table 4.

**Table 4:** Participant job role and practice characteristics

Practice number	Job role	Patient list size	Index of Multiple Deprivation (quintile)^**a**^
P01	Practice Manager	9887	6.14 (1st)
P02	Health Care Assistant	6841	46.02 (5th)
P03	Practice Manager	12,491	27.53 (4th)
P04	General Practitioner	7851	21.29 (3rd)
P05	Practice Nurse (manager)	6930	7.25 (1st)
P06	General Practitioner	8299	33.06 (4th)
P07	General Practitioner	5167	23.22 (4th)
P08	Practice Manager	8972	12.15 (2nd)

^a^Where 1 is the least deprived and 5 is the most deprived

Two domains within the CFIR were relevant to our data set. The first was Intervention Characteristics and themes emerged within constructs relating to the relative advantage of using the checklist its adaptability and overall design quality. The second domain was Outer setting and within the construct of Patient needs and resources the theme relating to a lack of capacity emerged. These are summarised in Table 5. Below we describe these emergent themes alongside exemplar quotes.

**Table 5:** Summary of CFIR Domains, Constructs and Emergent themes

CFIR Domain	Constructs	*Emergent themes*
**Intervention characteristics**	Relative advantage	*Staff engagement*
		*High level approach*
		*Prescribing safety*
		*Review existing systems*
		*Training staff*
	Adaptability	*Frequency of use*
	Design quality	*Quick to complete*
**Outer setting**	Patient needs and resources	*Lack of capacity*

### Intervention characteristics

This domain relates to the overall design, utility and usability of an intervention [20]. A number of constructs were identified in our data namely its Relative advantage, Adaptability and Design quality.

#### Relative advantage

This construct describes the stakeholder’s perception of the advantage of implementing the Prototype Safe-Systems Checklist (PSC) as opposed to maintaining existing practice. Any tool or instrument designed to improve safety of care can also improve aspects of care in other respects as patient safety and quality of care are so intrinsically linked. In terms of the advantages of using the PSC a number of themes emerged; staff described how they improved patient safety directly in terms of prescribing safety and enabling the review of existing systems, but also indirectly by using it to provide a framework to discuss patient safety with the broader practice team.

#### Staff engagement

Participants described how using the PSC indirectly benefitted patient safety by helping engage a range of staff. Although the tool was designed to be used by a single individual frequently, its completion would or could rely on other members of the practice team, helping raise awareness of patient safety.

“So we found it on several levels a really useful tool and not least, of course, patient safety, but in terms of actually being another vehicle to encourage cross-team understanding within the practice, as well.” Practice Manager, P01.

One Practice Manager felt that the document could be used to frame a discussion with GPs on whether policies and procedures were implemented as expected.

“…it’s quite straightforward, I’ll just run through everything with the GPs instead of saying ‘yes, we do this’… I mean you can have policy and procedure and no-one can follow it.” Practice Manager, P06.

#### High level approach

The benefits of the high level approach adapted by the checklist as a way of immunising specific items against local or sporadic change were described.

“I think one of the things that’s hard … with the checklist, is… keeping it up to date as things change so fast in practice, but a lot of your sentences are quite high-level, so it means that it lasts…” Practice Manager, P03.

#### Prescribing safety

A number of participants commented on the benefits of using the tool to improve medication safety and one practice manager felt the section on medications was the most useful.

“The medication things I thought was probably the most useful section… they say the most errors in a general practice are made on medicines…” Practice Manager, P03.

The other area that the PSC appeared to be effective was at highlighting the non-collection of repeat prescriptions. One GP acknowledged how this item had raised awareness of the issue and a practice manager how it had encouraged them to discuss the issue with other members of the team.

“Non-collection of prescriptions, that’s the one that we found that we weren’t doing very well… because we’re moving to electronic prescribing in a couple of weeks’ time, we’ll look into that, that way…” GP, P02.

“The non-collection of prescriptions was good and that did encourage me to talk to the dispensing team – “what did they do with those?”” Practice Manager, P07.

#### Review existing systems

It was noted, how as a whole, the PSC provided the opportunity to look again at the safety of existing systems that due to familiarity might otherwise be overlooked.

“Actually, it gives you the chance to reflect that some of the things [we do] are a system and to think, ‘Oh, yes!’ Something like mail-handling is, like so embedded …we take 500 letters in… every day, scan them in, pass them round and whatever - that, you know, you can almost forget that that is a safe system.” Practice Manager P03.

#### Training staff

Another way in which the PSC may indirectly benefit patient safety is by its use as a training tool for clinicians in the early part of their career. One practice manager described how it presented a useful overview for inexperienced clinicians.

“One thing I thought it would be …a good training tool for, like, an overview...These things would be good for, like, GP registrars and things, like in training… it’s a good overview position.” Practice Manager, P03.

### Adaptability

The construct of Adaptability describes the degree to which an intervention can be tailored, refined or reinvented to better meet local needs [20]. The flexibility of the PSC in terms of how frequently it could be used emerged.

#### Frequency of use

There was no prescribed time interval in between using the PSC, meaning that practices could decide how often it could be used. One practice manager described how they might use the tool monthly..

“…If you’re doing it monthly, you’re more aware of the questions in your head, aren’t you, so it’ll become more of a routine. So, yes, I think it would [be monthly], in the long term.” Practice Manager, P04.

Another practice manager felt it would be usefully applied every 12 months to ensure systems were operating as safely as expected.

“I think once you’ve checked through it, it might be worth just going through it on an annual basis, just to make sure that you are doing these things….” Practice Manager, P06.

### Design quality

The construct of Design quality describes the perceptions of users of the quality of its design [20]. The primary design element which participants commented on was how straightforward it was to use.

#### Ease of use

The PSC was considered well-structured and easy to follow, which meant that it was quick and easy to use.

“I think because it is quite brief it’s quite a useful thing, just a pointer to go through it and make sure that these things are still being done as they should.” Practice Manager, P06.

### Outer setting

The domain of Outer setting relates to the influence of factors external to the design of the tool and the organisation [20], and the relevant construct in our analysis related to patient needs and resources.

### Patient needs and resources

This describes the extent to which the practice understands and is able to meet the needs of its patients [20]. Within this construct the emergent theme concerned the lack of capacity of practices to absorb additional work streams.

#### Lack of capacity

One factor that may inhibit its further use was the limited capacity, in terms of time and workload in primary care. Despite not knowing the length of time it would take to use the tool, a GP at one practice asked a part-time member of staff to be responsible for the tool because of concerns over their own lack of time.

“Because we were just totally snowed under, so I knew I wouldn’t have time to do this so I asked my colleague who only works part time and did that for me. So he’s… done the Safe Systems questionnaire.” – GP P02.

One practice manager was positive towards the PSC but cautioned that its future implementation might depend on the ability of practices to meet the twin pressures of time and resource.

“As much as I am a big fan of this tool, I think the two key issues are finding time and, if it involves any resources, is actually finding support for those resources because that’s always challenging in this day and age.” Practice Manager, P01.

## Discussion

### Main findings

We have described the creation of the prototype safe systems checklist (PSC) a new tool in patient safety developed for inclusion in the Royal College of General Practitioners hosted *Patient Safety Toolkit* [3] (Please see Additional file 1 for a final version of the PSC). The development involved gaining a structured consensus of priority issues gathered from an international panel of experts underpinned by published evidence. These became the draft list of 13 items from which nine items were selected and edited for clarity based on user feedback. The 9-item prototype was tested in general practice, and participating practices reported that they met the majority of the items; seven out of nine checklist items were answered ‘yes’ by seven of nine practices with only two items (follow-up of vulnerable patients after discharge and the non-collection of prescriptions) being answered no by two practices. When asked about their experience of the checklist participants spoke favourably of its brevity, the relevance of its content, and its ability to frame broader safety-based discussions across the practice team. However that limitations of capacity familiar across much of UK general practice might hinder its wider implementation.

### Strengths and limitations

The PSC has fulfilled the requirement for a tool that can quickly and easily provide valuable information on previously overlooked yet significant systems influencing primary care safety. Though the instrument described here is a prototype and yet to be fully validated its prima facie value has subsequently seen its successful incorporation into the patient safety toolkit [3]. The practices testing this instrument were located in one geographical area (North Staffordshire) yet their characteristics were reflective of national averages [32]. Drawing interviewees from this small pool of potential candidates that had used the checklist may have served to reduce response bias [33]. The ‘ceiling effect’ observed for some of the items [34] may be explained at least in part by the commitment of practices involved in the development of the Patient Safety Toolkit to improving patient safety.

### Specific findings

#### Relative advantage

##### Staff engagement

For any intervention to be adopted it is necessary that it is perceived to offer significant advantages over existing methods of work [20]. There is a tendency to see checklists as a summative ‘tick-box’ exercise in which a list of all ‘Yes’ answers is more important than any process of system maturation, yet checklists, like other quality or safety improvement interventions, can also be used for formative purposes [35, 36]. The PSC is designed to be used as part of formative learning and development exercises that take the needs and experiences of each practice as the starting point [37]. Successful quality improvement is facilitated by close team working within practices [38, 39] and by strategies that include the wider practice team [40], particularly where they enable practice staff to learn, work, and plan together with clear objectives [41, 42]. In relation to this staff described a number of areas where the PSC might be used and one of the most significant was its use in framing conversations across the practice team that reflect on the safety of practice systems and participants.

##### Prescribing safety

Each checklist item was precisely described to provide the opportunity to reflect on not only whether but how each item was achieved inhibiting the tendency to tick an item for the sake of compliance [34]. Of the items selected for inclusion in the PSC participants described how those relating to the non-collection of repeat prescriptions were of significant value acknowledging that existing systems lacked clarity in this area. The importance of the safety of medicines and prescribing in the era of polypharmacy has been widely described [42, 43] and is recognised as a key area of focus for the future of primary care safety [44].

##### Review of existing systems

Participants described how the PSC enables senior practice staff e.g. practice managers or GP partners to review existing systems equally it might support an audit of a particular area or process for example around the communication of results or non-collection of prescriptions, again promoting intra-practice discussion and collective learning.

#### Design quality

Participants described the ease of use of the PSC and the speed with which it could be completed. The PSC was designed to be quick and easy to use, requiring neither facilitation nor specialist training. Though suitable as a framework for wider practice discussion, the checklist does not require the practice team to meet simultaneously to be effective. This offers a complementary set of outputs to the other GP checklist tool in the public domain, the NHS Education for Scotland (NES) checklist which consists of 78 items designed to help practices prepare for CQC or similar inspections [45].

#### Adaptability

The frequency with which the PSC can be used is dependent on the preferences of individual practices with our participants expecting benefits whether used monthly or annually. It’s been recognised previously that in healthcare checklists should be monitored to avoid over-burdening staff [46] and general practice in particular is a field in which practitioners have been shown to be over-worked [47]. This adaptability may also be increased by creating a digital version which was not available at the time of testing yet electronic versions of similar general practice tools has resulted in enhanced utility [30].

#### Outer setting

Despite the ease and flexibility of its use the longer term implementation of the PCS remained subject to constraints on resources which could impact on additional work streams [48]. This reflects the tension that persists in UK primary care between improving quality and safety amidst a sustained increase in demand [47] being met by a workforce that continues to lose experienced staff [49].

## Conclusions

Successfully assessing quality and safety requires a mixture of subjective and objective approaches, [50, 51] and the PSC appears to offer a valuable objective lens through which to reflect on subjective procedures and actions within individual practices. It is important that checklists produced for general practice have their origin in safety factors relevant to their environment and are designed for use in the real world. The PSC meets our goal of highlighting relevant yet less obvious safety factors in general practice processes and has now become an integral part of the RCGP Patient Safety Toolkit.

## Supplementary information

**Additional file 1.**

**Additional file 2.**

## Data Availability

The datasets used and/or analysed during the current study are available from the corresponding author on reasonable request.

## Abbreviations

CQBT: Canadian Quality Book of Tools
CQC: Care Quality Commission
PSC: Prototype Concise Safe-Systems Checklist
EM: East Midlands
GM: Greater Manchester
NIHR-SPCR: National Institute for Health Research School for Primary Care Research
NES: NHS Education for Scotland
NHS: National Health Service
NS: North Staffordshire
PST: Patient Safety Toolkit
PMCPA: Prescription Medicines Code Of Practice Authority

## References

[1] Kachalia A (2016). Legal and policy interventions to improve patient safety. Circulation.

[2] Wachter RM (2010). Patient safety at ten: unmistakable progress, troubling gaps. Health Aff (Millwood).

[3] Practitioners, R.C.o.G. Patient Safety Toolkit for General Practice. [cited 2016 March 8]; Available from: http://www.rcgp.org.uk/clinical-and-research/patient-safety.aspx. Accessed 18 Mar 2016.

[4] Organisation, T.W.H. Safe Surgery Checklist. [cited 2016 March 8]; Available from: http://www.who.int/patientsafety/safesurgery/ss_checklist/en/. Accessed 18 Mar 2016.

[5] Marcelli D (2015). Implementation of a quality and safety checklist for haemodialysis sessions. Clin Kidney J.

[6] Haynes AB (2009). A surgical safety checklist to reduce morbidity and mortality in a global population. N Engl J Med.

[7] Lorincz CY, et al. Research in ambulatory patient safety 2000–2010: a 10-year review. Chicago: American Medical Association; 2011.

[8] Bowie P (2015). Participatory design of a preliminary safety checklist for general practice. Br J Gen Pract.

[9] Society, T.M.P. Clinical Risk Assessments for General Practice. [cited 2015 July 31]; Available from: http://www.medicalprotection.org/uk/education-and-events/workshops/clinical-risk-self-assessments/clinical-risk-self-assessments-for-gp-practices. Accessed 30 July 2015.

[10] Bell, B., et al., The Development and Testing of the NIHR-SPCR Patient Safety Toolkit for General Practices in England (Part 2). 2020.

[11] Spencer R, Campbell SM (2014). Tools for primary care patient safety: a narrative review. BMC Fam Pract.

[12] Bell BG (2014). Tools for measuring patient safety in primary care settings using the RAND/UCLA appropriateness method. BMC Fam Pract.

[13] Care Quality Commission. Available from: https://www.cqc.org.uk/. Accessed 1 Aug 2019.

[14] Health and Safety Executive. Health and social care services legislation. [cited 2015 July 31]; Available from: http://www.hse.gov.uk/healthservices/index.htm. Accessed 31 July 2015.

[15] Ricci-Cabello I (2015). Measuring experiences and outcomes of patient safety in primary care: a systematic review of available instruments. Fam Pract.

[16] Bell BG (2016). Safety climate in English general practices: workload pressures may compromise safety. J Eval Clin Pract.

[17] Practice, E.S.f.Q.a.S.i.F. Quality Book of Tools. Available from: https://equip.woncaeurope.org/tools/quality-book-tools. Accessed 1 Aug 2019.

[18] Robinson OC (2014). Sampling in interview-based qualitative research: a theoretical and practical guide. Qual Res Psychol.

[19] Jamshed S (2014). Qualitative research method-interviewing and observation. J Basic Clin Pharm.

[20] Damschroder LJ (2009). Fostering implementation of health services research findings into practice: a consolidated framework for advancing implementation science. Implement Sci.

[21] Gov.UK (2015). English indices of deprivation.

[22] Campbell SM, Chauhan U, Lester H (2010). Primary medical care provider accreditation (PMCPA): pilot evaluation. Br J Gen Pract.

[23] Casalino LP (2009). Frequency of failure to inform patients of clinically significant outpatient test results. Arch Intern Med.

[24] Dumay AC, Haaker TI (2010). The electronic locum record for general practitioners: outcome of an evaluation study in the Netherlands. Int J Med Inform.

[25] Xyrichis A, Lowton K (2008). What fosters or prevents interprofessional teamworking in primary and community care? A literature review. Int J Nurs Stud.

[26] Porcelli PJ, Waitman LR, Brown SH (2010). A review of medication reconciliation issues and experiences with clinical staff and information systems. Appl Clin Inform.

[27] Nassaralla CL (2007). Implementation of a medication reconciliation process in an ambulatory internal medicine clinic. Qual Saf Health Care.

[28] Anthony D, et al. In: Henriksen K, et al., editors. Re-engineering the hospital discharge: an example of a multifaceted process evaluation, in advances in patient safety: from research to implementation (Concepts and Methodology), vol. 2. Rockville: Agency for Healthcare Research and Quality (US); 2005. 21249814

[29] Mir C (2009). Impact of a computerized physician order entry system on compliance with prescription accuracy requirements. Pharm World Sci.

[30] Eguale T (2010). Enhancing pharmacosurveillance with systematic collection of treatment indication in electronic prescribing: a validation study in Canada. Drug Saf.

[31] Avery AJ (2005). Identifying and establishing consensus on the most important safety features of GP computer systems: e-Delphi study. Inform Prim Care.

[32] PublicHealthEngland (2020). National general practice profiles.

[33] Krosnick JA (1999). Survey research. Annu Rev Psychol.

[34] Hood C (2006). Gaming in targetworld: the targets approach to managing British public services. Public Adm Rev.

[35] Burian BK (2018). More than a tick box: medical checklist development, design, and use. Anesth Analg.

[36] Campbell SM, Grol M, Dautzenberg P (2004). External accountability for primary care, in Quality Management in Primary Care, R.

[37] Chevalier JM, Buckles DJ (2013). Participatory action research: theory and methods for engaged inquiry.

[38] Stevenson K (2001). Features of primary health care teams associated with successful quality improvement of diabetes care: a qualitative study. Fam Pract.

[39] Berwick DM (2003). Improvement, trust, and the healthcare workforce. BMJ Qual Safety.

[40] Ferlie EB, Shortell SM (2001). Improving the quality of health care in the United Kingdom and the United States: a framework for change. Milbank Q.

[41] Campbell SM (2002). Implementing clinical governance in English primary care groups/trusts: reconciling quality improvement and quality assurance. Qual Safety Health Care.

[42] Pedros C (2014). Prevalence, risk factors and main features of adverse drug reactions leading to hospital admission. Eur J Clin Pharmacol.

[43] Guthrie B (2015). The rising tide of polypharmacy and drug-drug interactions: population database analysis 1995-2010. BMC Med.

[44] NHSImprovement (2019). Patient Safety in Primary Care.

[45] Comission CQ (2010). Essential Standards of Quality and Safety, Guidance about compliance.

[46] Hales BM, Pronovost PJ (2006). The checklist- a tool for error management and performance improvement. J Crit Care.

[47] Association, B.M (2015). Quality First: Managing workload to deliver safe patient care.

[48] Litchfield I (2018). Influences on the adoption of patient safety innovation in primary care: a qualitative exploration of staff perspectives. BMC Fam Pract.

[49] BMA (2019). BMA responds to data on NHS vacancies, GP workforce and GP appointments.

[50] Greenhalgh T, Heath I (2010). Measuring quality in the therapeutic relationship-part 1: objective approaches. Qual Safety Health Care.

[51] Greenhalgh T, Heath I (2010). Measuring quality in the therapeutic relationship-part 2: subjective approaches. Qual Safety Health Care.

