# Supplementary material for: Development of the prototype concise safe systems checklist tool for general practice
Source: BMC Health Serv Res. 2020 Jun 16;20:544. doi: 10.1186/s12913-020-05396-y (PMC7296969; doi:10.1186/s12913-020-05396-y)
Supplement: Supplementary file 1 — Additional file 1. [file 12913_2020_5396_MOESM1_ESM.docx]

**Appendix 1 – Final Prototype Checklist**

|  |  | Yes | No | N/A | Any changes required? |
| --- | --- | --- | --- | --- | --- |
| 1. | All incoming clinical information is seen by nominated members of the team trained (or with relevant clinical experience) to deal appropriately with this information before the information is filed in the patient’s record. |  |  |  |  |
| 2. | Where incoming clinical information requires follow-up or diarised activity this is recorded in the patient’s record and acted upon |  |  |  |  |
| 3. | Where a clinician decides it is indicated, the patient (or where appropriate the patient’s representative) is informed of abnormal investigation results in an appropriately and timely manner and this contact is documented in the patient’s record. |  |  |  |  |
| 4. | The practice keeps a log of minor operations containing all the following information   - Date/patient’s name - Procedure performed - Who performed the operation and who assisted - Consent taken - Any complications - Specimen sent for Histology Y/N - Patient informed of result |  |  |  |  |
| 5. | Up-to-date information on practice policies, procedures and local facilities/services is provided to guide all temporary clinical staff (including GP registrars). |  |  |  |  |
| 6. | Non-collection of prescriptions is monitored or followed-up and is a trigger for review and audit in partnership with local pharmacies |  |  |  |  |
| 7. | Vulnerable patients discharged from hospital are followed-up by a member of the clinical team within 1 month |  |  |  |  |
| 8. | The indication for all repeat medications is coded within the electronic record (excluding topical preparations) |  |  |  |  |
| 9. | All staff are trained to make safe use of the prescribing elements of the clinical IT system which are relevant to their role |  |  |  |  |
